# NEIGHBORHOOD CONDITIONS AND SELF-NEGLECT IN LATER LIFE: LONGITUDINAL EVIDENCE FROM THE NSHAP

**DOI:** 10.1093/geroni/igz038.1025

**Published:** 2019-11-08

**Authors:** Laura Upenieks, James Iveniuk, Markus H Schafer

**Affiliations:** 1 University of Toronto, Toronto, Canada, Canada; 2 Wellesley Institute, Toronto, Ontario, Canada

## Abstract

Self-neglect includes persistent inattention to personal hygiene and the conditions of one’s immediate living environment and is known to be associated with an increased risk of mortality among older adults. Although previous studies have shown that many individual factors predict self-neglect, neighborhood characteristics have received much less attention. Extant research has yet to consider connections between the conditions of one’s neighborhood and self care over time. Using nationally representative longitudinal data from the National Social Life, Health, and Aging Project (NSHAP), we consider several features of neighborhood context in later life, including self-reported perceptions of neighborhood cohesion and neighborhood danger, neighborhood disorder (measured by interviewer ratings), and concentrated neighborhood disadvantage (using census data). Adjusting for individual-level factors (including social connection, physical and cognitive health, and demographics), results from both lagged dependent variable and cross-lagged panel models find higher levels of neighborhood disorder to be associated with higher self-neglect scores (measured by interviewer ratings) over time. Social cohesion, perceived neighborhood danger, and collective efficacy were not associated with self-neglect when controlling for neighborhood disorder. These findings suggest that improving neighborhood disorder may be an effective approach for self-neglect prevention in later life

